# Energy transport pathway in proteins: Insights from non-equilibrium molecular dynamics with elastic network model

**DOI:** 10.1038/s41598-018-27745-y

**Published:** 2018-06-22

**Authors:** Wei Bu Wang, Yu Liang, Jing Zhang, Yi Dong Wu, Jian Jun Du, Qi Ming Li, Jian Zhuo Zhu, Ji Guo Su

**Affiliations:** 1 Key Laboratory for Microstructural Material Physics of Hebei Province, College of Science, Yanshan University, Qinhuangdao, 066004 China; 2 Beijing Institute of Biological Products Co., Ltd, Beijing, 101111 China; 3 Beijing Key Lab of Digital Plant, Beijing Research Center for Information Technology in Agriculture, Beijing, 100097 China

## Abstract

Intra-molecular energy transport between distant functional sites plays important roles in allosterically regulating the biochemical activity of proteins. How to identify the specific intra-molecular signaling pathway from protein tertiary structure remains a challenging problem. In the present work, a non-equilibrium dynamics method based on the elastic network model (ENM) was proposed to simulate the energy propagation process and identify the specific signaling pathways within proteins. In this method, a given residue was perturbed and the propagation of energy was simulated by non-equilibrium dynamics in the normal modes space of ENM. After that, the simulation results were transformed from the normal modes space to the Cartesian coordinate space to identify the intra-protein energy transduction pathways. The proposed method was applied to myosin and the third PDZ domain (PDZ3) of PSD-95 as case studies. For myosin, two signaling pathways were identified, which mediate the energy transductions form the nucleotide binding site to the 50 kDa cleft and the converter subdomain, respectively. For PDZ3, one specific signaling pathway was identified, through which the intra-protein energy was transduced from ligand binding site to the distant opposite side of the protein. It is also found that comparing with the commonly used cross-correlation analysis method, the proposed method can identify the anisotropic energy transduction pathways more effectively.

## Introduction

Energy transport through specific pathways in proteins enables the communication between different parts of the molecule, which is thought to play important roles in many functional processes of proteins. Especially, the signaling between the distant allosteric and active sites may facilitate the long-range allosteric regulation of protein functions^1–5^. However, how to effectively identify the energy transport pathway between remote sites in protein tertiary structure remains a challenging problem in the research field of protein structure-function relationship.

Molecular dynamics (MD) simulation is an effective method to investigate the dynamical properties of proteins in atomistic detail^6,7^, which has been used to explore the signaling pathway in protein structures^8,9^. Ghosh and Vishveshwara performed MD simulations for methionyl-tRNA synthetase and the cross-correlation of motions between different residues was evaluated, which were then combined with the protein structure network analysis to elucidate the communication pathways between distant functional sites in the protein^10^. Kong and Karplus proposed a method, named as interaction correlation analysis, to identify the signaling pathways of proteins, in which the interaction energy correlations between all residue pairs were calculated based on the equilibrium MD simulations^11^. Ishikura and Yamato developed a theoretical method based on the long-time equilibrium MD simulations to analyze the pathways of inter-residue energy flow between different regions in proteins. In this method, the energy conductivity was evaluated by the time-correlation function of the inter-residue energy flux, which was numerically computed from the long MD simulation trajectories^12^. However, strictly speaking, the process of intra-protein energy transport is a non-equilibrium behavior, and therefore many studies utilized non-equilibrium MD simulations to investigate the transfer of energy in proteins. Ota and Agard proposed a non-equilibrium MD simulation method, called as anisotropic thermal diffusion, to detect the intramolecular signaling pathways. In their method, the probed residues or atoms are coupled to a higher temperature, serving as the energy source, whereas the temperature couplings for other atoms in the protein are turned off. Then, non-equilibrium MD simulations were performed and the signaling pathways were observed by detecting the propagation of the root mean square deviation for the residues in the protein^13^. Sharp and Skinner developed a pump-probe MD simulation method, in which the selected residues or atoms were excited with a set of oscillating forces and the energy transduction pathways were revealed by using Fourier transform of the atomic motions in the protein^14^. Stock group developed several methods of non-equilibrium MD simulations to study energy transport in molecules, where the selected atoms were perturbed from the ground-state to the excited-state to mimic the ultraviolet excitation and infrared vibration pumping experiments, or the selected atoms were heated by higher temperature to generate an energy flow in molecules. Then, based on the non-equilibrium MD simulation trajectories, a non-equilibrium correlation function was used to evaluate the cumulative response of the residues to the energy source^4,15–18^. However, for the equilibrium and non-equilibrium MD simulations, the signals are usually submerged in the noise, and some subtle analysis methods are required to achieve a better signal-to-noise ratio, such as simulation at the super-low temperature, comparison with the control simulations, and so on.

Besides the MD simulation, another commonly used method to investigate the dynamical properties of proteins is the normal mode analysis of the elastic network model (ENM)^19–22^. ENM describes the protein structure as an elastic network, in which the interactions between residues are simplified as springs. Because the inter-residue interactions are harmonic, the partition function and the thermodynamical properties of the protein can be analytically calculated. Many studies have shown that such a simple model is sufficient to account for many experimental data, such as the X-ray crystallographic temperature factors, the order parameters derived from NMR relaxation experiments, and the protection factors for H/D exchange experiments^23,24^. ENM can also reproduce MD simulation data but does not require the high computational cost of MD simulation^23–25^. ENM has been proved in many applications to be a simple yet effective method for investigating large-scale conformational transitions, allosteric motions, equilibrium fluctuation of residues, decomposition of domains for proteins, as well as identification of key residues and refinement of low-resolution structural data^19–34^. However, at present, most of the application studies for ENM are focus on exploring the dynamical properties of the equilibrium state. In the present work, a non-equilibrium dynamics method was proposed based on ENM to simulate the energy transduction, which is a non-equilibrium process, in proteins.

In our method, the selected residue was perturbed by assigning a displacement from its equilibrium position, which serves as the energy source. After that, the propagation of the perturbation was simulated by non-equilibrium dynamics in the normal modes space of ENM. The simulation results were then transformed from the normal modes space to the Cartesian coordinate space to monitor the intra-protein energy transduction process. The functionally key residues involved in energy propagation were identified as those dynamically coupled with the perturbed residue, which form the signaling pathway in the protein. In the present work, the proposed method was applied to myosin and the third PDZ domain of PSD-95 as case studies. Myosin is an important allosteric protein that transforms chemical energy provided by the hydrolysis ATP into force and movement along actin^35,36^. In this study, a residue in the ATP binding site was perturbed and the specific signaling pathway responsible for the allosteric regulation of the protein functions was identified. PSD-95, as a member of the PDZ domain protein family, plays important roles in regulating protein-protein interactions associated with synapses of the central nervous system^37^. It has been found that the residue His372 is responsible for the specificity of ligand binding^11,13,14,38^. In this work, the residue His372 was perturbed and then the signaling pathway from this residue to the remote functional sites was revealed by our proposed method. Our prediction results are consistent with the available experimental and simulation data.

## Methods

### Gaussian network model

Gaussian network model (GNM)^19,21–24^, one type of the ENM, describes the protein tertiary structure as an elastic network, in which each residue is simplified as a node represented by its C_α_ atom and the interactions between residues are simplified as springs. If the distance between two residues is less than the cutoff value (7.0 Å is adopted in this work), these two residues are considered to have interactions between them, and thus they are connected by a spring. Otherwise, there is no spring between them. In this model, the force constant is identical for all the springs. Considering all the harmonic interactions between residues, the potential energy of the system can be written as

1 $$V=\frac{\gamma }{2}\sum _{i,j}^{N}{({\rm{\Delta }}{R}_{ij})}^{2}=\frac{\gamma }{2}{\rm{\Delta }}{R}^{T}{\rm{\Gamma }}{\rm{\Delta }}R$$

where *γ* is the force constant of the springs, Δ*R*_*ij*_ is the displacement of the distance between the *i*^*th*^ and *j*^*th*^ residues, {Δ*R*} is the N-dimensional column vector of the fluctuations of all the residues, N is the number of residues in the protein, the superscript *T* denotes the transpose of the column vector, and Γ is the *N* × *N* dimensional Kirchhoff matrix whose elements are expressed as

2 $${{\rm{\Gamma }}}_{ij}=\{\begin{array}{cl}-\,1 & if\,i\ne j\,and\,{R}_{ij}\le {r}_{c}\\ 0 & if\,i\ne j\,and\,{R}_{ij} > {r}_{c}\\ -\,\sum _{j,j\ne i}^{N}{{\rm{\Gamma }}}_{ij} & if\,i=j\end{array}$$

where *R*_*ij*_ is the distance between the *i*^*th*^ and *j*^*th*^ residues, and *r*_*c*_ is the cutoff distance whose value is set to be 7.0 Å.

The normal modes of the motion for the system can be calculated by decomposing the Kirchhoff matrix Γ as

3 $${\rm{\Gamma }}=U{\rm{\Lambda }}{U}^{T}$$

where *U* is an orthonormal matrix whose columns are the eigenvectors of the Kirchhoff matrix Γ, and Λ is a diagonal matrix of the eigenvalues Λ_*ii*_(1 ≤ *i* ≤ *N*) of the matrix Γ. The value of the first eigenvalue is zero, and correspondingly the first eigenvector represents the overall translational motion of the system. In the present work, only the intra-protein motions are meaningful, therefore, the first eigenvector with zero eigenvalue is removed from the calculation.

According to the theory of statistical physics, the mean-square fluctuation of the *i*^*th*^ residue is given by

4 $$\langle {({\rm{\Delta }}{R}_{i})}^{2}\rangle =\frac{3{k}_{B}T}{\gamma }{[{{\rm{\Gamma }}}^{-1}]}_{ii}=\frac{3{k}_{B}T}{\gamma }\sum _{k=2}^{N}\frac{{U}_{ik}{U}_{ik}}{{{\rm{\Lambda }}}_{kk}}$$

The cross-correlation fluctuations between the *i*^*th*^ and *j*^*th*^ residues can be calculated by

5 $$\langle {\rm{\Delta }}{R}_{i}\cdot {\rm{\Delta }}{R}_{j}\rangle =\frac{3{k}_{B}T}{\gamma }{[{{\rm{\Gamma }}}^{-1}]}_{ij}=\frac{3{k}_{B}T}{\gamma }\sum _{k=2}^{N}\frac{{U}_{ik}{U}_{jk}}{{{\rm{\Lambda }}}_{kk}}$$

The normalized cross-correlation between the *i*^*th*^ and *j*^*th*^ residues is given by

6 $${C}_{ij}=\frac{\langle {\rm{\Delta }}{R}_{i}\cdot {\rm{\Delta }}{R}_{j}\rangle }{{[\langle {({\rm{\Delta }}{R}_{i})}^{2}\rangle \cdot \langle {({\rm{\Delta }}{R}_{j})}^{2}\rangle ]}^{1/2}}$$

### The GNM-based non-equilibrium dynamics method for monitoring the intra-protein energy transport pathway

According to Newton’s law, the dynamical equation of the system can be written as

7 $$M\frac{{d}^{2}({\rm{\Delta }}R)}{d{t}^{2}}=M\frac{{d}^{2}R}{d{t}^{2}}=-\,\nabla V=-\,\nabla (\frac{\gamma }{2}{\rm{\Delta }}{R}^{T}{\rm{\Gamma }}{\rm{\Delta }}R)=-\,\gamma {\rm{\Gamma }}{\rm{\Delta }}R$$

where *M* is a diagonal matrix whose diagonal elements are the mass of each residue, *R* is the position vector of the residues in the protein, and ∇ is the gradient operator. In ENM, the mass is set to be identical for all the residues, which is represented as *m*, then the dynamical eq. (7) can be further expressed as

8 $$\frac{{d}^{2}({\rm{\Delta }}R)}{d{t}^{2}}=-\,\frac{\gamma }{m}{\rm{\Gamma }}{\rm{\Delta }}R$$

Then, the solution of the dynamical eq. (8) is

9 $${\rm{\Delta }}R(t)=\sum _{k=2}^{N}{U}_{k}{A}_{k}\,\cos ({\omega }_{k}t+{\phi }_{k})$$

where *U*_*k*_ is the *k*^*th*^ eigenvector of the matrix Γ, ${\omega }_{k}=\sqrt{\frac{\gamma }{m}{{\rm{\Lambda }}}_{kk}}$ represent the angular frequency of the *k*^*th*^ normal mode, and *A*_*k*_ and *φ*_*k*_ are respectively the amplitude and initial phase of the *k*^*th*^ normal mode, which are determined by the initial condition of the system.

When the selected residue, supposed as the *m*^*th*^ residue, was perturbed as the energy source, a displacement from the equilibrium position is assigned to this residue. The amplitude of the displacement is set to 1. Then, the initial column vector of the residue fluctuations at the time *t* = 0 can be written as

10 $${\rm{\Delta }}R(t=0)=(\begin{array}{c}\begin{array}{c}0\\ \vdots \end{array}\\ 1\\ \begin{array}{c}\vdots \\ 0\end{array}\end{array})$$

where the *m*^*th*^ element of the fluctuation vector Δ*R* is 1, and all the other elements are 0. In eq. (9), it is assumed that all the *φ*_*k*_ = 0, then the initial fluctuation vector can be projected onto all the normal modes as

11 $${\rm{\Delta }}R(t=0)=\sum _{k=2}^{N}{U}_{k}{A}_{k}$$

The initial amplitude for each normal mode can be determined as

12 $${A}_{k}= < {U}_{k}|{\rm{\Delta }}R(t=0) > =({U}_{1k},\,\cdots ,\,{U}_{mk},\,\cdots \,{U}_{Nk})(\begin{array}{c}\begin{array}{c}0\\ \vdots \end{array}\\ 1\\ \begin{array}{c}\vdots \\ 0\end{array}\end{array})={U}_{mk}$$

The time-evolution of each normal mode is ${U}_{k}{A}_{k}\,\cos \,{\omega }_{k}t={U}_{k}{U}_{mk}\,\cos \,{\omega }_{k}t$. Considering all the normal modes, the residue fluctuations as a function of time can be calculated by

13 $${\rm{\Delta }}R(t)=\sum _{k=2}^{N}{U}_{mk}{U}_{k}\,\cos \,{\omega }_{k}t$$

It should be noted that here ${\rm{\Delta }}R(t)={({\rm{\Delta }}{R}_{1}(t),{\rm{\Delta }}{R}_{2}(t),\cdots ,{\rm{\Delta }}{R}_{N}(t))}^{T}$, which is the N-dimensional column vector of the fluctuations of all the residues. In this work, the energy transport pathway was investigated by monitoring the time-evolution residue fluctuations, and the residues with relative large fluctuation are identified as the key residues involved in energy transport.

### Data availability

All data generated or analysed during this study are included in this published article, and the source code of our method is available from the corresponding author upon request.

## Results and Discussion

### The signaling pathways in myosin revealed by the GNM-based non-equilibrium dynamics method

**Figure 1:**
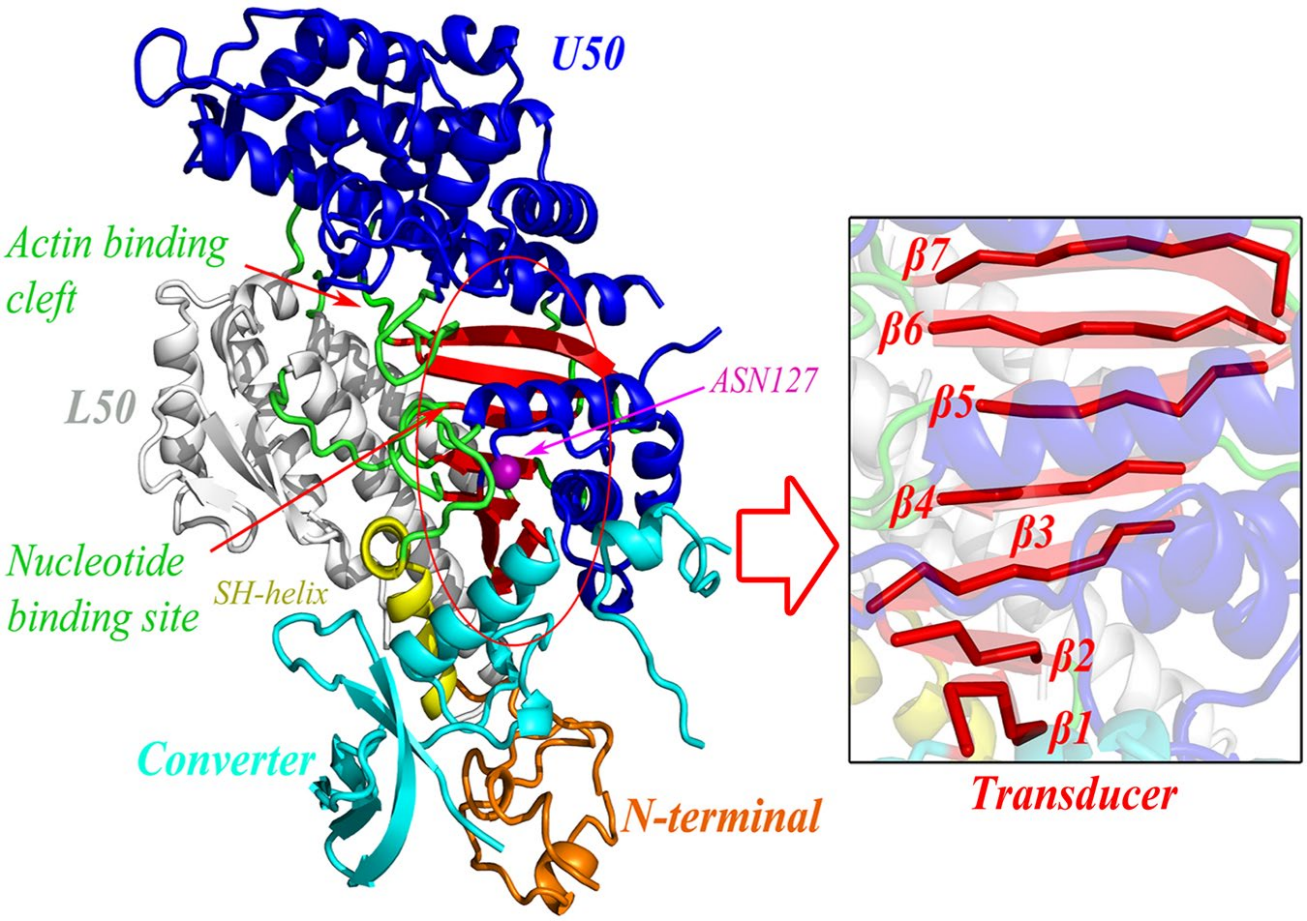
The tertiary structure of myosin (PDB code: 1MMA). The four subdomains of myosin are displayed in different colors: orange, the N-terminal subdomain; blue and red, the U50 subdomain; gray, the L50 subdomains; cyan and yellow, the converter subdomain. The seven β-strands β1–β7, which form the transducer region, are highlighted in the right enlarged figure. The SH1 helix is marked in yellow color. The locations of the nucleotide binding site and the actin binding cleft are respectively marked in the figure. The perturbed residue Asn127 is highlighted in purple ball.

Myosin is an ATP-driven motor protein that transforms the chemical energy of ATP into mechanical force and movement along actin. Myosin plays essential roles in a wide range of biological processes, such as muscle contraction, cell division, shape changing of the cell, gating of the force-activated ion channel, transporting of the vesicles, structural changing of neurons, and so on^35,36,39,40^. The structure of myosin is composed four subdomains, i.e., the N-terminal subdomain, the upper (U50) and lower (L50) 50 kDa subdomains, and the converter subdomain (also called the lever arm), as shown in Fig. 1. X-ray crystallographic and biochemical experiments for different conformations of myosin have indicated that there exist allosteric communications linking the nucleotide binding site, the actin binding cleft, and the movements of the lever arm^35,36,40–43^. The crystal structures of different myosin states have shown that without the binding of ATP, myosin binds strongly to actin with a closed 50 kDa cleft between the catalytic site and the actin binding site, and the lever arm is in the down position. The binding of ATP to the catalytic site results in the opening of 50 kDa cleft and the release of myosin from actin. Then, the hydrolysis of ATP induces the lever arm moving from its down position to up position^42–45^. The binding and hydrolysis of ATP is allosteric coupled with the open-closed conformational transition of 50 kDa cleft and the movement of the lever arm. In the present work, the proposed GNM-based non-equilibrium dynamics method was used to investigate how the signaling propagates from the nucleotide binding site to the distant regions in myosin. The structure of the near-rigor state of the Dictyostelium discoideum myosin (PDB code: 1MMA) was used in our calculation.

**Figure 2:**
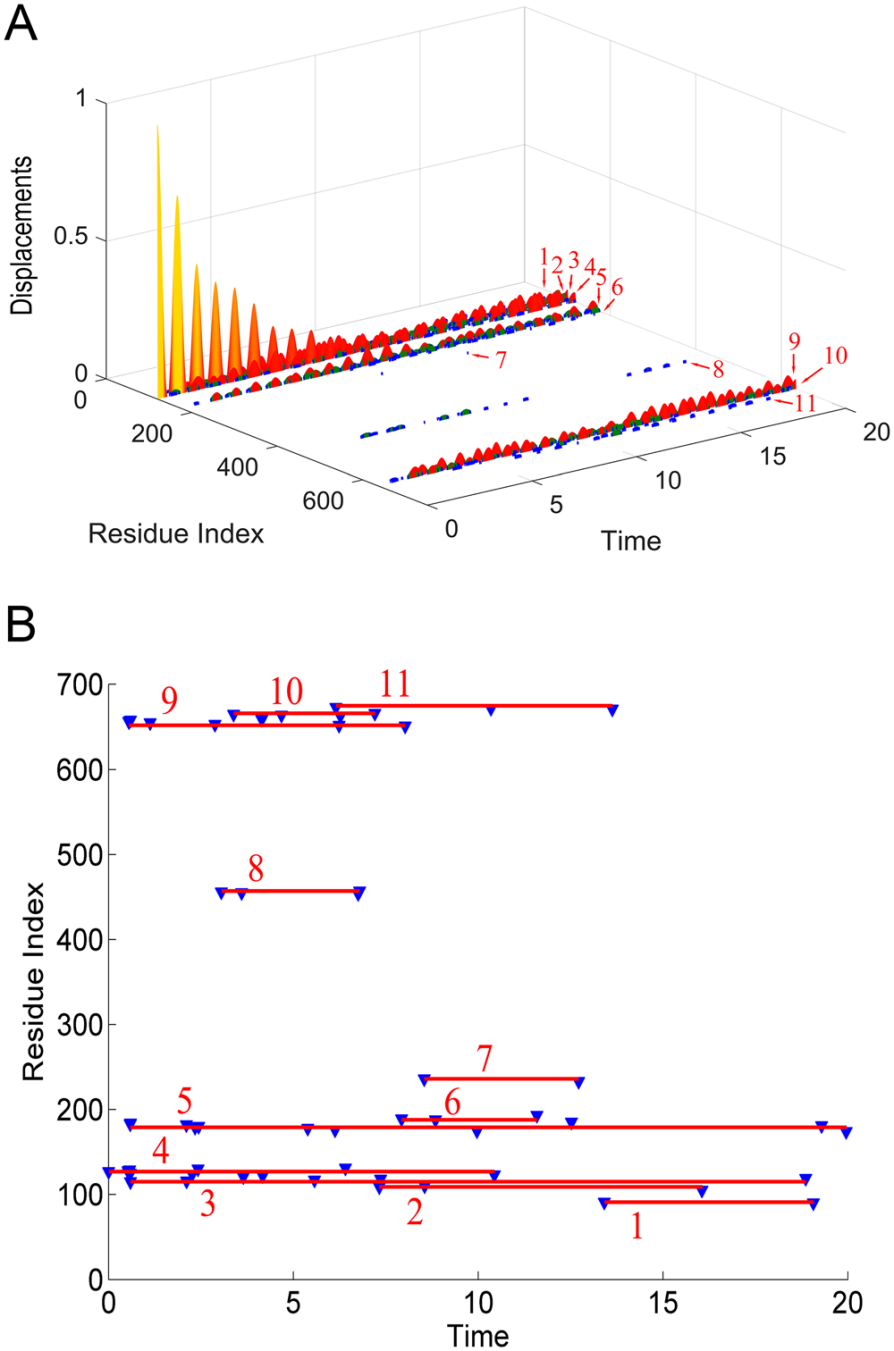
The residues involved in the intra-protein energy propagation for myosin. (**A**) The time-evolution of residue fluctuations in response to the perturbation of the residue Asn127. The residue clusters with relative large amplitude of fluctuations are grouped into 11 clusters marked by the numbers 1–11. (**B**) The excited residues, whose fluctuation amplitude is larger than 0.01, versus their excitation times. These excited residues are grouped into 11 clusters, where each cluster is marked by a red line with the cluster number. In this figure, the unit of time is $\sqrt{\frac{m}{\gamma }}$.

**Figure 3:**
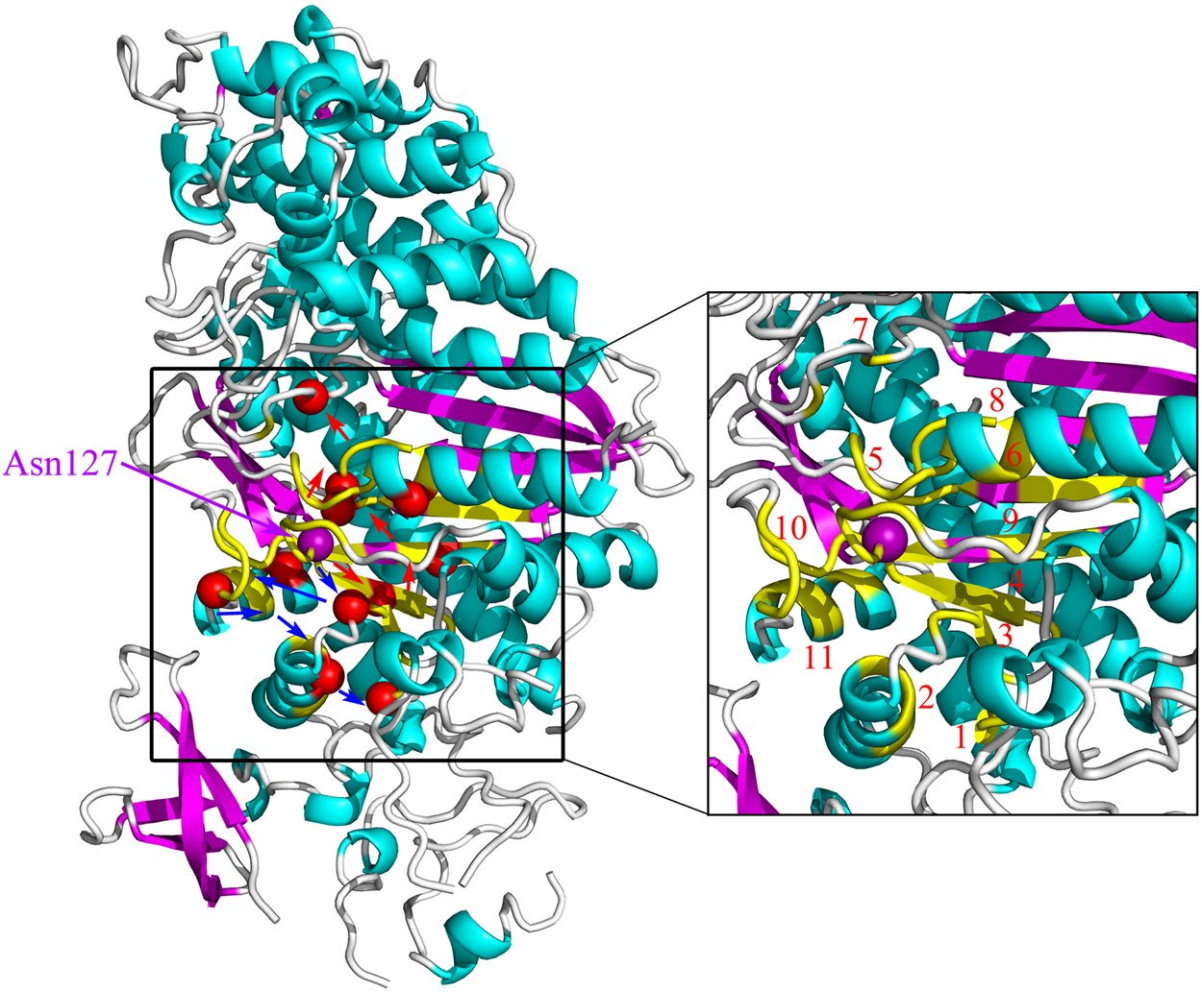
The key residues involved in energy transduction mapped onto the tertiary structure of myosin (PDB code: 1MMA). In this figure, the 56 energy-excited residues are shown in yellow color, which are grouped into 11 residue clusters. The central residues for these 11 residue clusters are marked by red balls. The signaling pathway from the ATP binding site to the distant 50 kDa cleft is indicated by red arrows, and the other pathway connecting the ATP binding site to the converter subdomain is denoted by blue arrows. The energy-excited region is enlarged in the right figure, in which the 11 residue clusters are marked by the numbers 1–11.

The residue Asn127 in the ATP binding site, which is directly involved in the nucleotide binding, was chosen as the perturbation site. According to our proposed method introduced in the “Methods” section, an initial displacement was assigned to this residue as the energy source, and then the energy propagation process was investigated by the ENM-based non-equilibrium dynamics calculation. The time-evolution of the amplitude of residue fluctuations for the system in response to the perturbation is shown in Fig. 2A. It is found that there are several clusters of residues that have relative large amplitude of fluctuations during energy propagation, which implies that these residues are involved in the signaling transduction. Figure 2B displays the residues with squared fluctuation larger than 0.01, along with the time when these residues are excited. From Fig. 2A,B, it is found that there are 56 energy-excited residues that distinctly involved in the intra-protein energy transduction. In order to display the results more intuitively, these 56 key residues involved in energy transduction are mapped onto the tertiary structure of myosin, which are exhibited in yellow color, as shown in Fig. 3. It is found that these excited residues are mainly located on the β strands and the associated connecting loops in the transducer subdomain. Considering that the functional sites, i.e. the 50 kDa cleft and the converter, are located at the top and bottom of the transducer, the energy propagation between β strands and between different loops (i.e. along the direction perpendicular to the β strands of the transducer) is thought to be responsible for the transduction of the functional signals. Therefore, in the present study, the excited residues located on the same β strand or loop are grouped into a residue cluster, and we only focused on the signaling pathway between different clusters. According to this definition, these 56 excited residues can be grouped into 11 clusters centered at residues Met91 (including Asp90 and Met91), Arg109 (including Asn105, Arg109 and Tyr110), Ile115 (including Ile115-Ser119), Val124 (including Leu121-Arg131), Gly179 (including Ser174-Leu176 and Thr178-Lys185), Asn188 (including Asn188, Thr189 and Ile193), Ser236 (including Asn233 and Ser236), Gly457(including Asp454-Gly457), Phe652 (including His651-Val653 and Cys655-Asn659), Lys666 (including Leu663-Lys666), and Gln675 (including Val671, Val672 and Gln675). These clusters are marked by the numbers 1–11 in Fig. 2A,B, and the central residues for the residue clusters are marked by red balls in Fig. 3. The residues of clusters 1–11 are respectively located on the loop at the interface between the N-terminal subdomain and the lever arm (cluster 1), helix 5 (cluster 2), β1 (cluster 3), β2 (cluster 4), β4 and the connecting P-loop (cluster 5), helix 9 connected to the perturbation residue (cluster 6), β6 and the connecting switch I (cluster 7), β5 and the connecting switch II (cluster 8), β3 (cluster 9), the loop connecting to SH1 helix (cluster 10), and SH1 helix in the converter subdomain (cluster 11), as shown in the enlarged sub-graph in Fig. 3. β1–β6, as well as switch I and II, belong to the so-called transducer of myosin, in which β5 and β6 are located at the 50 kDa cleft. It is found that many of these energy-excited residues are distant from the energy source Asn127 whereas many residues nearer the energy source are not excited, which implies that the energy can be allosterically transduced to the remote functional site through specific pathways. Our calculation results show that the transducer, the SH1 helix, and the bottom of the 50 kDa cleft are involved in the transfer of the energy from ATP binding site, which form the energy transduction pathway. Experimental studies have also indicated that the transducer region is crucial for the chemo-mechanical transduction, where the binding and hydrolysis of ATP results in the mechanical distortion of the transducer and then the conformational change of the transducer induces the opening of 50 kDa cleft and the swing of the lever arm^35,42,46,47^. There exist distinct allosteric couplings among the nucleotide binding site, the 50 kDa cleft and the converter subdomain. Our calculation results are well consistent with the experimental observations.

In order to investigate how the perturbation energy propagates among these clusters of residues, the sequence of energy excitation of these residue clusters was obtained from Fig. 2B. For each residue cluster, the time when any residue in the cluster was firstly excited was considered to be the excitation time of that cluster. As shown in Fig. 2B, the excitation times for the 11 residue clusters are 13.420 (cluster 1), 7.327 (cluster 2), 0.591 (cluster 3), 0.001 (cluster 4), 0.590 (cluster 5), 7.931 (cluster 6), 8.543 (cluster 7), 3.052 (cluster 8), 0.553 (cluster 9), 3.385 (cluster 10) and 6.150 (cluster 11), respectively. The unit of time is $\sqrt{\frac{m}{\gamma }}$. According to the chronological order of the excitation of these residue clusters, two pathways of energy transduction can be identified, as indicated by arrows in Fig. 3. One pathway is Asn127 → β2 (cluster 4) → β3 (cluster 9) → β4 and the connecting P-loop (cluster 5) → β5 and the connecting switch II (cluster 8) → β6 and the connecting switch I (cluster 7), as denoted by red arrows in Fig. 3. Along this pathway, the energy was transferred from the ATP binding site to the distant 50 kDa cleft, which may contribute to the regulation of the actin binding and dissociation. The other signaling pathway is Asn127 → β1 (cluster 3) → the loop connecting to SH1 helix (cluster 10) → SH1 helix in the converter subdomain (cluster 11) → helix 5 (cluster 2) → the loop at the interface between the N-terminal subdomain and the lever arm (cluster 1), as indicated by blue arrows in Fig. 3. Through this pathway the perturbation energy at the ATP binding site is transduced to the converter subdomain, which may facilitate the produce of force and movement of the lever arm. Many experimental studies have indicated that the transducer region in myosin mediates the allosteric couplings among the nucleotide binding site, the 50 kDa cleft and the converter subdomain^35,36,40–43,46,47^. However, the detailed signaling pathways among these sites are still largely unknown. Our study provided the signaling pathways and the associated key residues for the allosteric communications between the functional sites of myosin. It should be noted that the excitation time for the residues within the cluster span a broad range, as shown in Fig. 2B, which implies that it takes a relative long time for the energy transduction within residue cluster. Here the residue clusters mainly corresponds to the secondary structures in the protein. Our calculation results indicate that the signaling transduction within the secondary structure is usually slower than the signaling between the secondary structures.

It should be mentioned that in our study the initial phase of each normal mode is set to zero, which means that the initial velocity of each normal mode is zero and thus the initial velocity of the perturbed residue is zero. We also investigated whether the initial velocity of the perturbed residue has influences on the energy transduction pathway. Therefore, both an initial displacement and an initial velocity are assigned to the perturbed residue. It should be noted that the initial velocity will result in non-zero initial phases of the normal modes. The detailed theoretical derivation of our method for non-zero initial velocity is given in the Supplementary material. The calculation results are displayed in Supplemental Fig. 1 and Supplemental Table 1 in the Supplementary material, which were compared with the case of zero initial velocity. It is found that the initial velocity has little influences on the identification of the excited residues as well as the excitation times of these key residues. The same energy transduction pathways were obtained both for the zero and non-zero initial velocity of the perturbed residue. (see the detailed discussions provided in the Supplementary material).

### The signaling pathways in the third PDZ domain of PSD-95 revealed by the GNM-based non-equilibrium dynamics method

**Figure 4:**
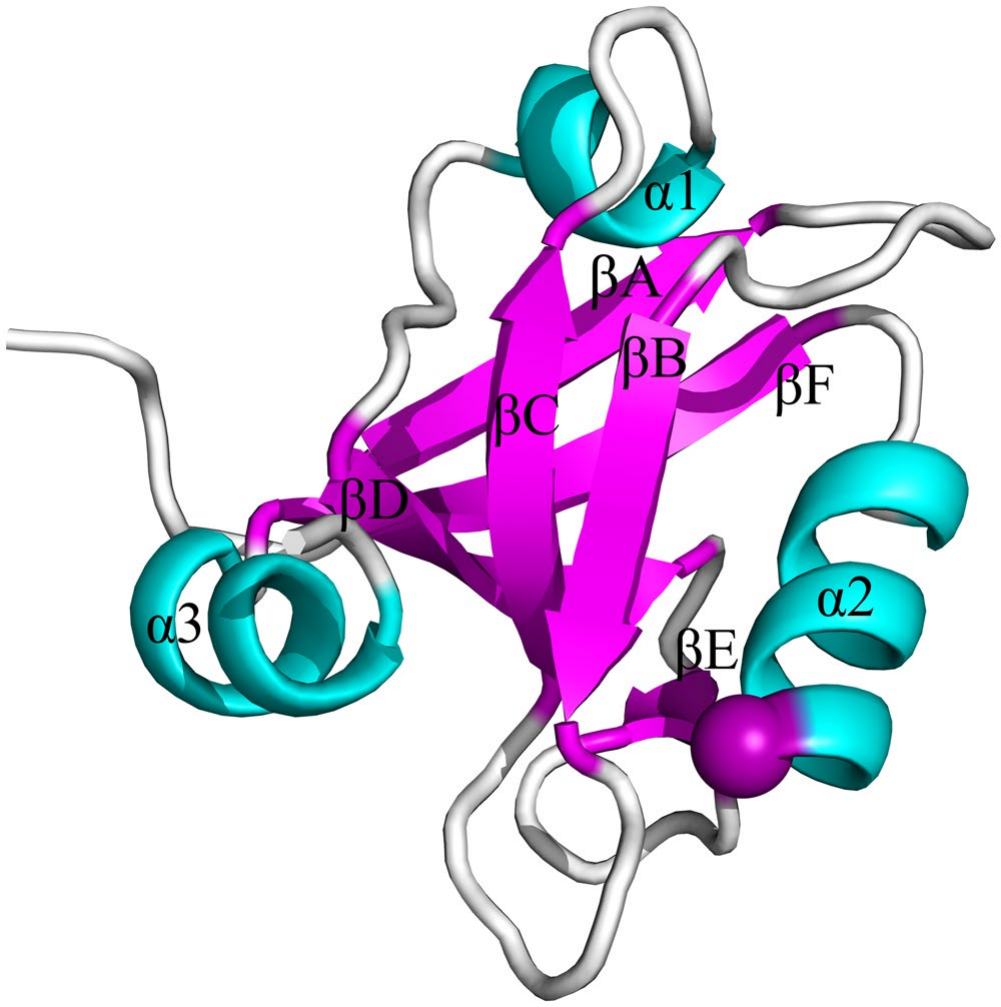
The tertiary structure of the third PDZ domain of PSD-95 (PDB code: 1Be9). The six β strands βA–βF and three helix α1–α3 are marked in this figure. The perturbed residue His372 is highlighted in purple ball.

The third PDZ domain (PDZ3) of PSD-95 (PDB code: 1BE9) plays an important role in mediating the signaling of the central nervous system, which can bind to the C-terminus of ion channels and form signaling complexes at cellular membranes^37,48,49^. The PDZ3 domain of PSD-95 has 97 residues, which is composed of six β strands and three α helices, as shown in Fig. 4. Comparing with the canonical PDZ domain that has six β strands and two α helices, α3 in the C-terminus is unique to PDZ3. Several theoretical and experimental studies have indicated that the allosteric signaling can be transferred in the PDZ3 domain from the ligand binding site to the remote region through specific residue-residue interactions^13,14,50^. In the ligand binding site, the residue His372 has proved by experiments to be responsible for the specificity of ligand binding, which directly interacts with the ligand through hydrogen bonds^11,13,14,37,38^. In this study, the residue His372 was perturbed as the energy source, and then the transduction pathway of the energy was predicted by using the proposed ENM-based non-equilibrium dynamics method.

**Figure 5:**
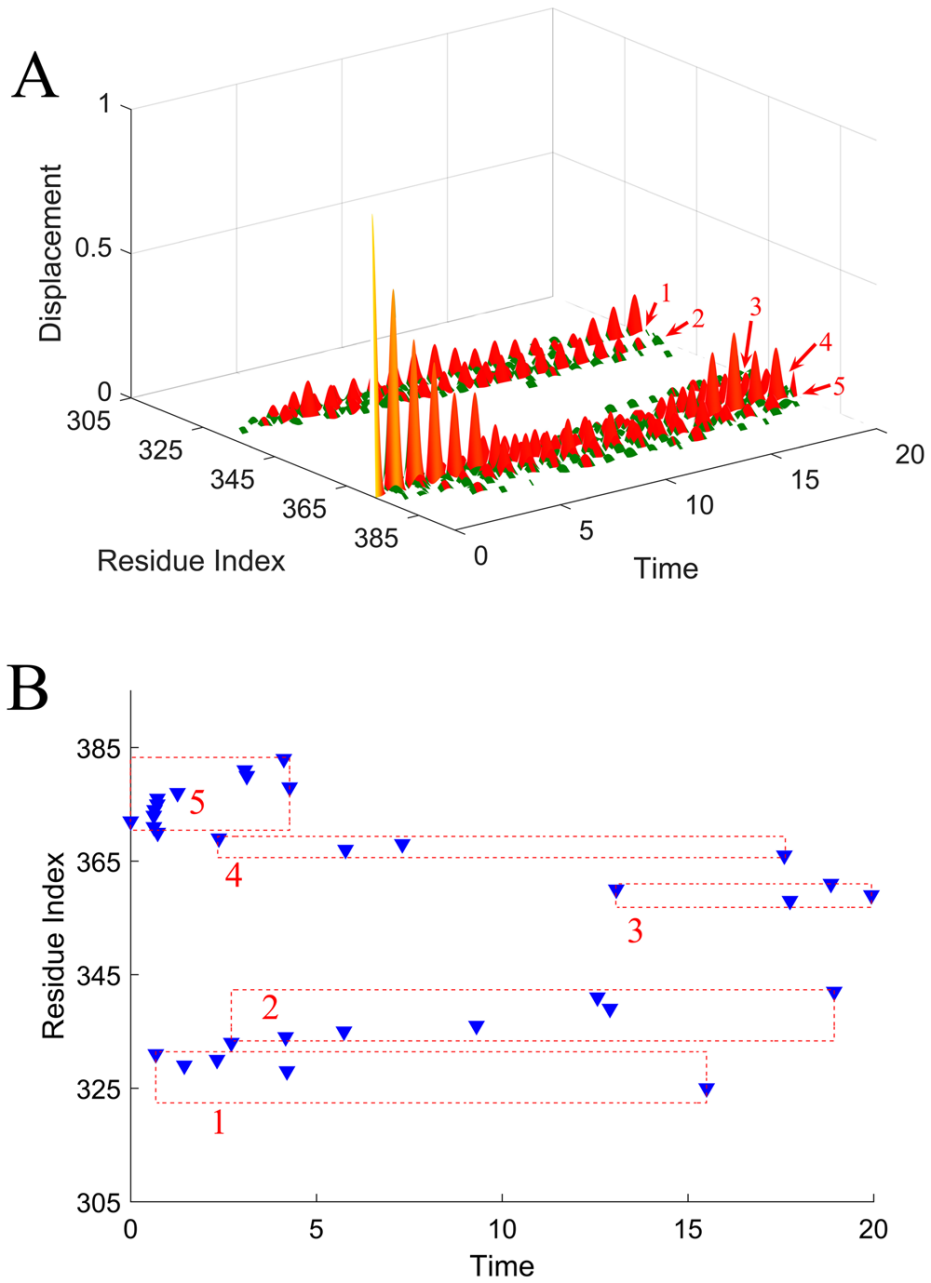
The residues involved in the intra-protein energy propagation for the PDZ3 domian. (**A**) The time-evolution of residue fluctuations for myosin in response to the perturbation of the residue His372. The residue clusters with relative large amplitude of fluctuations are grouped into 5 clusters marked by the numbers 1–5. (**B**) The excited residues, whose squared fluctuation is larger than 0.01, versus their excitation times. These excited residues are grouped into 5 clusters, where each cluster is marked by a red box with the cluster number. In this figure, the unit of time is $\sqrt{\frac{m}{\gamma }}$.

**Figure 6:**
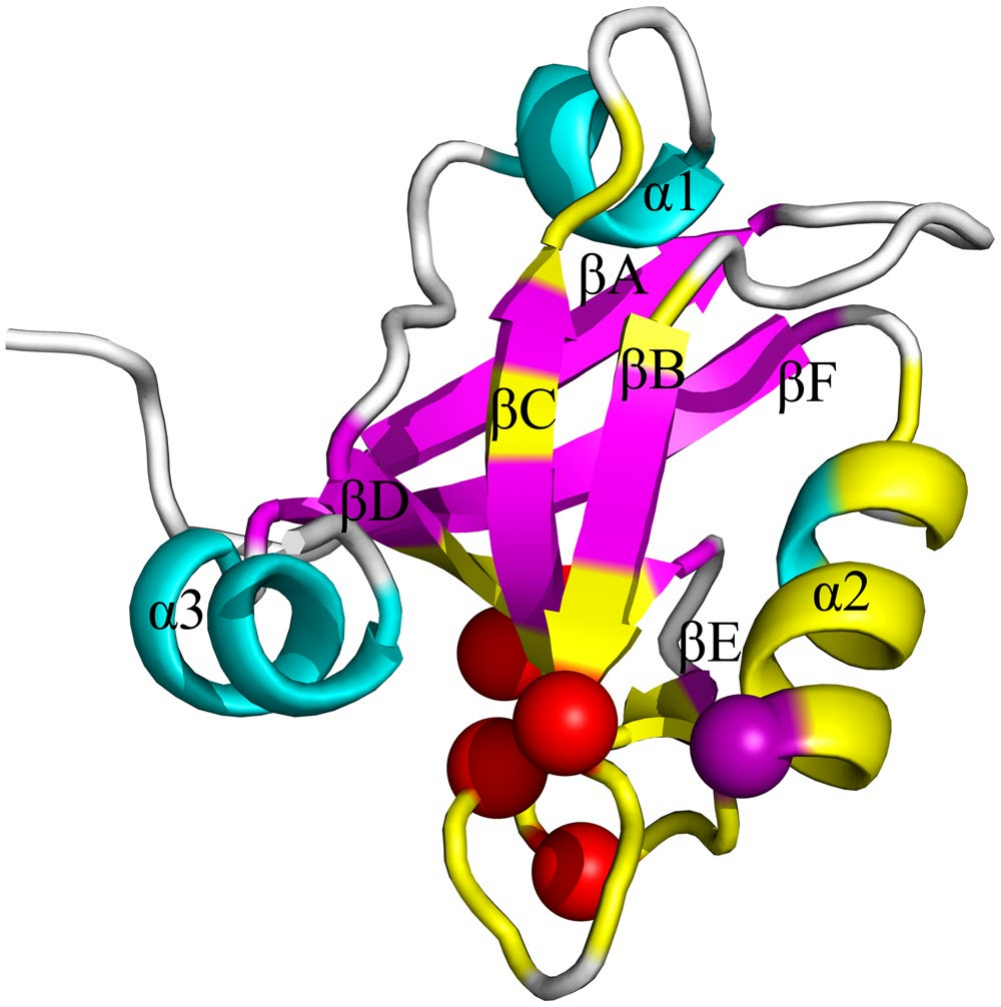
The key residues involved in energy transduction mapped onto the tertiary structure of the PDZ3 domain of PSD-95 (PDB code: 1BE9). In this figure, the 32 energy-excited residues are shown in yellow color, which are grouped into 5 residue clusters. The central residues for these 5 residue clusters are marked by purple and red balls. The signaling pathway in this protein is indicated by red arrows.

The time-evolution of the amplitude of residue fluctuations for the system in response to the perturbation of His372 is shown in Fig. 5A. The residues with fluctuation amplitude larger than 0.015, along with the time when these residues are excited, are displayed in Fig. 5B. It is found that there are 32 energy-excited residues that involved in intra-protein energy transduction. Figure 6 displays these 32 residues mapped onto the tertiary structure of PDZ3 domain, in which the locations of these energy-excited residues are shown in yellow color. The residues located on the same β strand, loop or α helix are grouped into one cluster, and we only focused on the energy transduction pathway between different clusters. According to their location on the protein structure, these 32 excited residues are grouped into 5 clusters, i.e., Phe325, Ile328, Gly329, Gly330, Glu331 (cluster 1), Gly333, Glu334, Gly335, Ile336, Ser339, Ile341, Leu342 (cluster 2), Gln358, Ile359, Leu360, Ser361 (cluster 3), Asp366, Leu367, Arg368, Asn369 (cluster 4), and Ala370, Ser371, His372, Glu373, Gln374, Ala375, Ala376, Ile377, Ala378, Lys380 Asn381, Gly383 (cluster 5). The cluster number is marked in Fig. 5A,B, and the central residues of these clusters are highlighted by red balls and labeled by the cluster numbers in Fig. 6. These residue clusters are located at the βB (cluster 1), the βC (cluster 2), the βD (cluster 3), the βE and the connecting loop between βE and α2 (cluster 4), α2 along with the loop between α2 and βF (cluster 5). The sequence of energy excitation of these residue clusters was obtained from Fig. 5B to investigate how the perturbation energy propagates among these clusters of residues. The excitation times for the 5 residue clusters are 0.667 (cluster 1), 2.719 (cluster 2), 13.056 (cluster 3), 2.380 (cluster 4) and 0.001 (cluster 5), respectively, as marked by red circles in Fig. 6. The unit of time is $\sqrt{\frac{m}{\gamma }}$. According to the chronological sequence of residue excitations, the signaling pathway can be identified as His372 → βB → the βE and the connecting loop between βE and α2 → βC → βD, as indicated by red arrows in Fig. 6. Through this pathway, the intra-protein energy was transduced from ligand binding site to the opposite side of the protein. Several computational^13,14,50^ and experimental studies^50–54^ have showed that the dynamic coupling of the residues between different β strands in the core of PDZ domain plays critical roles for the transduction of allosteric signals. Our predicted signaling pathway is consistent with the results obtained from the evolutionary analysis of sequences, MD simulations, and the mutation experiments.

### The anisotropy of the intra-protein signaling propagation

**Figure 7:**
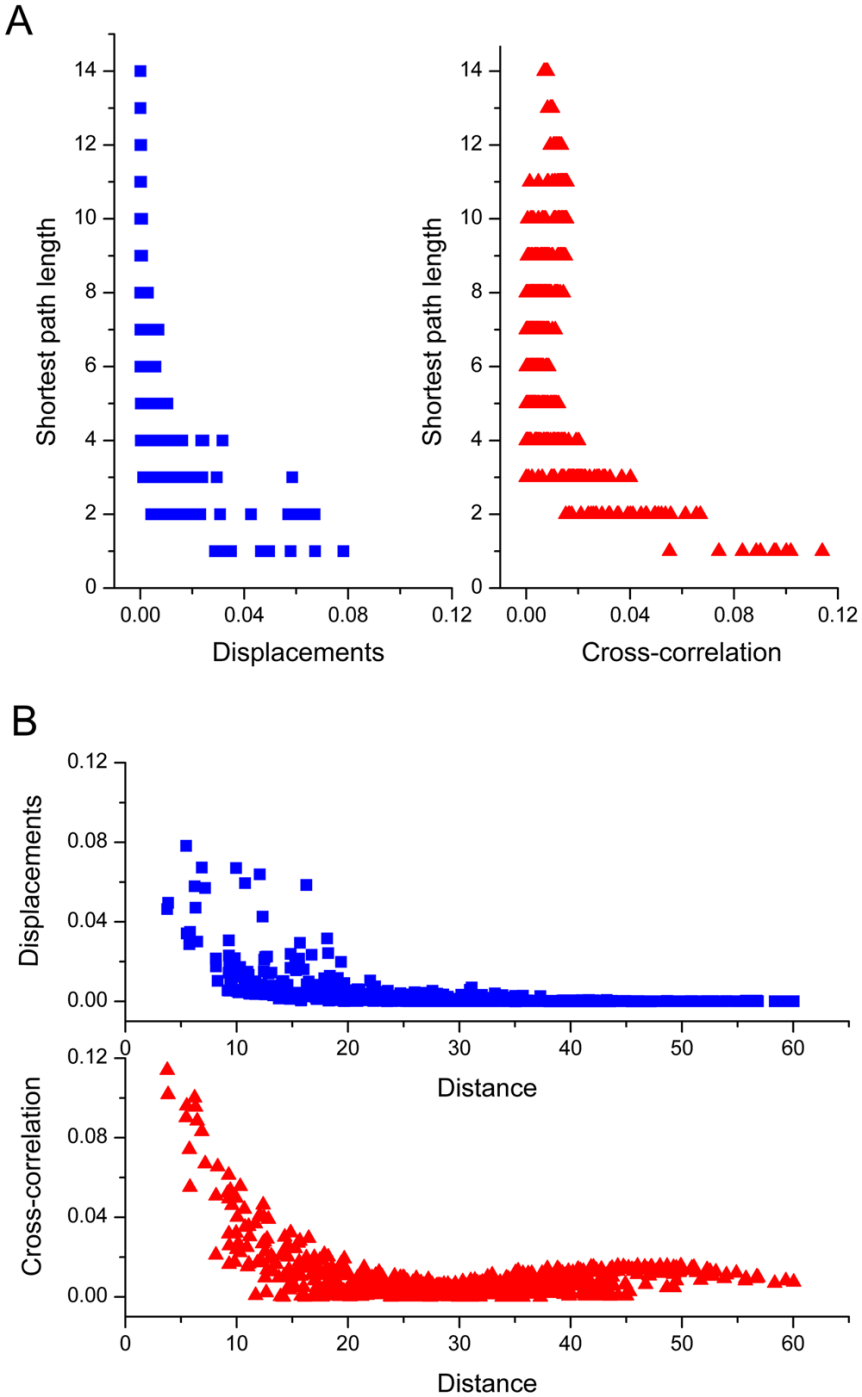
The anisotropy of the intra-protein signaling propagation in myosin revealed by our method, compared with the results obtained by the normalized cross-correlation analysis method. (**A**) Left: The amplitude of residue fluctuation calculated by our method versus the shortest path length of that residue to the perturbed residue Asn127; Right: The normalized cross-correlation between each residue and Asn127 versus its shortest path length to Asn127. (**B**) Upper: The fluctuation amplitude of the residues as a function of their distance to the energy source Asn127; Lower: The normalized cross-correlation between each residue and Asn127 versus its distance to Asn127.

Our calculation results discussed in the above section show that both for myosin and PDZ3 domain many residues distant from the energy source were excited, whereas the residues nearer to the energy source may not be excited. The result indicates that the energy transduction is anisotropic. In order to more intuitively illustrate the anisotropy of the energy propagation in myosin, the amplitude of residue fluctuation versus the shortest path length of that residue to the energy source is shown in the left sub-graph of Fig. 7A. The shortest path length for each residue to the energy source was calculated by the Dijkstra algorithm^55^. If the energy propagation is isotropic, the residues with the same shortest path length should have similar fluctuation amplitude and the fluctuation amplitude should decrease along with the increase of the shortest path length. However, the left sub-graph in Fig. 7A shows that the residues with the same shortest path length span a wide range of fluctuation amplitude, and the anti-correlation between the residue fluctuation amplitude and its shortest path length is not pronounced. This result implies that the intra-protein energy propagation is distinctly anisotropic in myosin. The upper sub-graph in Fig. 7B displays the fluctuation amplitude of the residues as a function of their distance to the energy source for myosin. If the energy diffusion is isotropic, an inverse relationship between the fluctuation amplitude of the residues and their distance to the energy source should be observed, in which the residue fluctuation decreases with the increase of the distance. However, the inverse relationship is not appeared in the upper sub-graph of Fig. 7B. These above results indicate that the intra-protein energy propagation is anisotropy and there exists specific signaling pathways between different regions in myosin, which may facilitate the allosteric regulation of protein functions. The similar calculation results were obtained for the PDZ3 domain (data not shown).

### Comparison with the results obtained by the normalized cross-correlation analysis of the equilibrium dynamics

**Figure 8:**
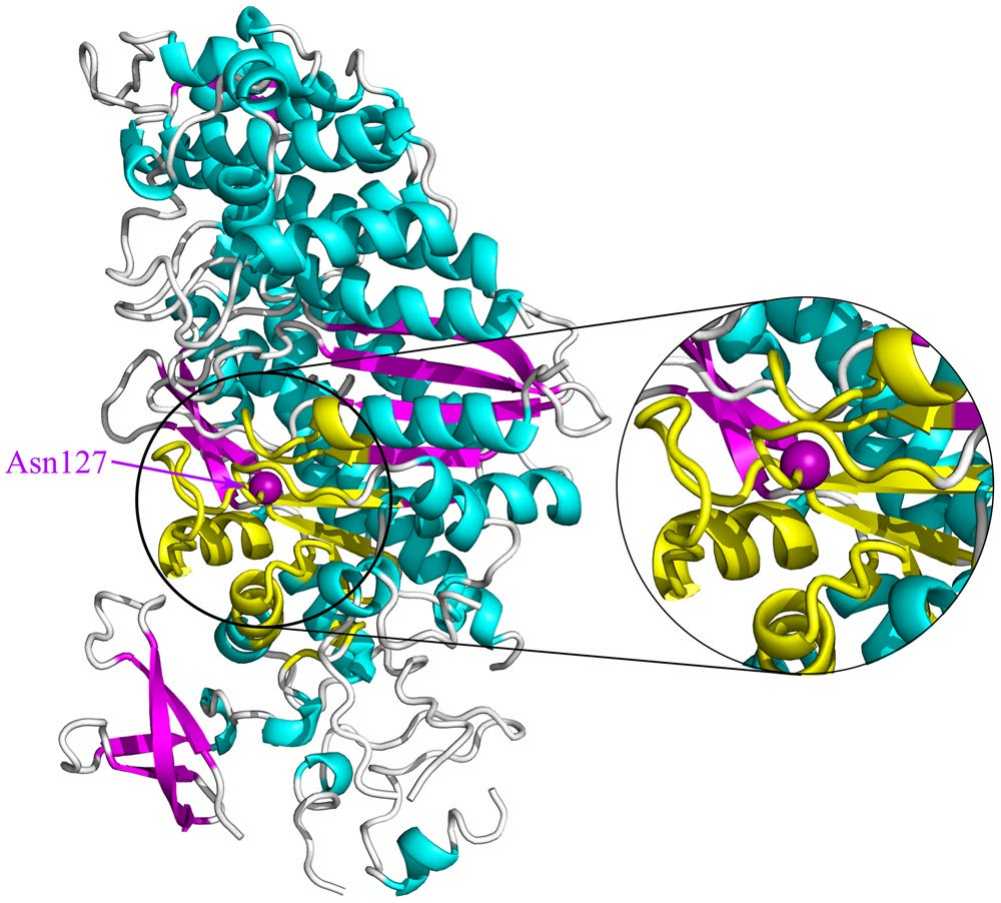
The first 10% residues mostly dynamically coupled with Asn127, revealed by the normalized cross-correlation analysis method, were mapped on to the tertiary structure of myosin. These residues dynamically coupled with Asn127 are displayed in yellow color.

The cross-correlation analysis of the residue fluctuations in the equilibrium state is a commonly used method to investigate the dynamical couplings between different regions in protein tertiary structure^11,56–58^. In this work, the ENM-based normalized cross-correlation analysis method was also applied for myosin to illustrate the difference between this method and our proposed method. According to eq. (6) discussed in the “Methods” section, the normalized cross-correlation between each residue and the residue Asn127 in myosin was calculated. The first 10% residues that are mostly dynamically coupled with Asn127 were mapped on to the tertiary structure of myosin, as shown by yellow color in Fig. 8. Comparing Fig. 8 with Fig. 3, it is found that the residues identified by the cross-correlation analysis method distribute more isotropically around Asn127, which indicates that this method does not work well in detecting the anisotropic signaling pathway. In order to illustrate the isotropic property of the cross-correlation analysis method, the normalized cross-correlation between each residue and Asn127 versus its shortest path length to Asn127 is displayed in the right sub-graph of Fig. 7A. The normalized cross-correlation between each residue and Asn127 versus its distance to Asn127 is displayed in the lower sub-graph of Fig. 7B. This figure exhibits that the value of cross-correlation decreases almost linearly as the distance increases, implying that the cross-correlation values distribute isotropically. Comparing the sub-graphs in Fig. 7A,B, it is found that the key residues identified by our method distribute more anisotropically than the cross-correlation analysis method. These results imply that our method is more effective than the cross-correlation analysis method in revealing the anisotropic signaling pathway. For example, our method revealed that the perturbation energy in Asn127 transfers through β3 and β4 to the remote regions in β5 and β6, however β5 and β6 cannot be identified by the cross-correlation analysis method, as shown in Figs 3 and 8. The similar results were obtained for the PDZ3 domain of PSD-95 (data not shown). From a physical standpoint, our method is a non-equilibrium dynamics method theoretically applicable to study the non-equilibrium process of intra-protein energy transduction. Whereas, the cross-correlation between residue fluctuations only reflects the equilibrium dynamical properties, which may not suitable for detecting the signaling in the non-equilibrium process.

## Conclusion

Intra-molecular signaling between distant functional sites plays important roles in many functional processes of proteins. In the present work, a non-equilibrium dynamics method based on ENM was proposed to simulate the energy propagation process and identify the specific signaling pathways within proteins. In this method, a given residue was perturbed as the energy source, and then the propagation of the perturbation energy was calculated by non-equilibrium dynamics in the normal modes space of ENM. Then, the calculation results were transformed from the normal modes space to the Cartesian coordinate space to reveal the intra-protein energy transduction pathways.

Using the proposed method, the long-range signaling pathways between distant functional sites in myosin and PDZ3 domain of PSD-95 were successfully identified. For myosin, two signaling pathways were obtained: one is Asn127 → β2 → β3 → β4 → β5 → β6, and the other is Asn127 → β1 → the loop connecting to SH1 helix → SH1 helix → helix 5 → the loop at the interface between the N-terminal subdomain and the lever arm. The first pathway connects the nucleotide binding site to the distant 50 kDa cleft, which may contribute to the allosteric coupling between the binding of ATP the dissociation of actin. The second pathway mediates the energy transduction from the ATP binding site to the converter subdomain, which may facilitate the produce of force and movement of the lever arm. For the PDZ3 domain of PSD-95, the specific signaling pathway was identified as His372 → βB → the βE and the connecting loop between βE and α2 → βC → βD, through which the intra-protein energy was transduced from ligand binding site to the distant opposite side of the protein.

The calculation results of our method for myosin were compared with those obtained by the cross-correlation analysis method. It is found that the residues dynamically coupled with Asn127 identified by the cross-correlation analysis method are isotropically distributed around Asn127. The cross-correlation analysis method can reveal the equilibrium dynamical properties of proteins, but it is failed to identify the anisotropic pathways for the non-equilibrium energy transduction. In contrast, our method as a non-equilibrium dynamics method can effectively identify the long-range signaling pathways, and the pathways are highly anisotropic. Our method is generally applicable for different protein systems, and this study provides a starting point for the investigations of other proteins.

It should be mentioned that compared with the conventional MD simulation method, our proposed GNM-based non-equilibrium dynamics method is rigorous in theoretical deduction and simple in calculation. In the conventional MD method, the motion of atoms in protein system is simulated by numerically integrating Newton’s motion equations, and the dynamic properties of the system are calculated based on a large number of samplings in the conformational space. MD simulation method is time-consuming and easy to trap in local optimum, especially for larger protein systems. Besides that, in MD simulations the signals may be submerged in the noise and some subtle analysis methods usually need to be well-designed to improve the signal-to-noise ratio. In our proposed GNM-based method, the dynamic properties of protein systems can be calculated analytically, without long-time numerically simulations. Therefore, our method is computationally efficient and can be easily applied to large-size protein systems.

## Electronic supplementary material

Supplementary material
