# Supplementary material for: Energy transport pathway in proteins: Insights from non-equilibrium molecular dynamics with elastic network model
Source: Sci Rep. 2018 Jun 22;8:9487. doi: 10.1038/s41598-018-27745-y (PMC6015066; doi:10.1038/s41598-018-27745-y)
Supplement: Supplementary file 1 — Supplementary material [file 41598_2018_27745_MOESM1_ESM.pdf]

Energy transport pathway in proteins: Insights from non-equilibrium molecular dynamics with elastic network model

Wei Bu Wang, Yu Liang, Jing Zhang, Yi Dong Wu, Jian Jun Du, Qi Ming Li, Jian Zhuo Zhu, Ji Guo Su

The non-equilibrium dynamics method for non-zero initial velocity of the perturbed residue

In our study discussed in the main text, the initial phase of each normal mode is set to zero, which means that the initial velocity of each normal mode is zero and thus the initial velocity of the perturbed residue is zero. In order to investigate whether the initial velocity of the perturbed residue has influences on the energy transduction pathway, both an initial displacement and an initial velocity are assigned to the perturbed residue. It should be noted that the initial velocity will result in non-zero initial phases of the normal modes. The non-equilibrium dynamics method for non-zero initial velocity is given in this Supplementary material. Then, the calculation results were compared with the case of zero initial velocity.

According to the theory of GNM discussed in the main text of the paper, the displacement and velocity of the residues as a function of time are given respectively by

$$\Delta R(t) = \sum_{k=2}^{N} U_k A_k cos(\omega_k t + \varphi_k) \quad (1)$$

$$v(t) = -\sum_{k=2}^{N} U_k A_k \omega_k sin(\omega_k t + \varphi_k) \quad (2)$$

where U_k is the kth eigenvector of the matrix Γ, $\omega_k = \sqrt{\frac{\gamma}{m}\Lambda_{kk}}$ represent the angular frequency of the kth normal mode, Λ_{kk} is the eigenvalue of the kth normal mode, and A_k and φ_k are respectively the amplitude and initial phase of the kth normal mode, which are determined by the initial condition of the system.

Then, a selected residue, supposed as the mth residue, was perturbed by assigning an initial displacement as well as an initial velocity to this residue. The amplitudes of both the displacement and the velocity are set to 1. This initial condition can be written as

$$\Delta R(t=0) = \begin{pmatrix} 0 \\ \vdots \\ 1 \\ \vdots \\ 0 \end{pmatrix} \text{ and } v(t=0) = \begin{pmatrix} 0 \\ \vdots \\ 1 \\ \vdots \\ 0 \end{pmatrix} \quad (3)$$

where the mth elements of both the fluctuation vector ΔR and the velocity v are 1, and all the other elements are 0. Substituting Equation (3) into Equations (1) and (2), the initial fluctuation and velocity vectors can be projected onto all the normal modes as

$$\Delta R(t=0) = \sum_{k=2}^{N} U_k A_k\, cos\varphi_k \quad (4)$$

$$v(t=0) = -\sum_{k=2}^{N} U_k A_k\, \omega_k sin\varphi_k \quad (5)$$

Based on Equations (4) and (5), we can get

$$A_k cos\varphi_k = \langle U_k | \Delta R(t=0) \rangle = (U_{1k}, \cdots, U_{mk}, \cdots U_{Nk}) \begin{pmatrix} 0 \\ \vdots \\ 1 \\ \vdots \\ 0 \end{pmatrix} = U_{mk} \quad (6)$$

$$-A_k\omega_k sin\varphi_k = \langle U_k | v(t=0)\rangle = (U_{1k}, \cdots, U_{mk}, \cdots U_{Nk})\begin{pmatrix}0\\ \vdots\\ 1\\ \vdots\\ 0\end{pmatrix} = U_{mk} \quad (7)$$

Then, according to Equations (6) and (7), the initial amplitude and phase for each normal mode can be determined as

$$A_k = \frac{U_{mk}}{\omega_k}\sqrt{1+\omega_k^2} \quad (8)$$

$$\varphi_k = arctan(-\frac{1}{\omega_k}) \quad (9)$$

The time-evolution of each normal mode is $U_k A_k \cos(\omega_k t + \varphi_k) = U_k \frac{U_{mk}}{\omega_k}\sqrt{1+\omega_k^2}\cos[\,\omega_k t + arctan(-\frac{1}{\omega_k})]$. Considering all the normal modes, the residue fluctuations as a function of time can be calculated by

$$\Delta R(t) = \sum_{k=2}^{N} U_k \frac{U_{mk}}{\omega_k}\sqrt{1+\omega_k^2}\, cos[\,\omega_k t + arctan(-\frac{1}{\omega_k})] \quad (10)$$

The calculation results for the non-zero initial velocity compared with the case of zero initial velocity

Based on Equation (10), the energy transport pathway was investigated by monitoring the time-evolution residue fluctuations, and the residues with relative large fluctuation are identified as the key residues involved in energy transport. Then, the calculation results for the non-zero initial velocity were compared with the case of zero initial velocity. The calculation results for myosin are shown in Supplemental Figure 1. This figure exhibits the excited residues, whose squared fluctuation larger than 0.010 (in the case of zero initial velocity) or 0.011(in the case of non-zero initial velocity), versus their excitation times. The blue triangles in Supplemental Figure 1 represent the 56 energy-excited residues identified by the method without initial velocity, and these residues are grouped into 11 clusters marked by the red lines along with the cluster numbers. When an initial velocity was assigned to the perturbed residue, 58 excited residues were identified, which are marked by the green crosses in Supplemental Figure 1. It is found that in the 58 excited residues, 56 residues are identical to those identified by the method without initial velocity. The other two residues are neighboring to these identical excited residues in the sequence. Our calculation results indicated that the initial velocity has negligible influences on the identification of the excited residues responsible for the intra-energy transduction.

In addition, the excitation times for most of the excited residues are nearly the same in these two cases, as shown in Supplemental Figure 1. In our paper, the time when any residue in the cluster was firstly excited was considered as the excitation time of each cluster. According to this definition, the excitation times of these 11 excited residue clusters were determined in the two cases with zero and non-zero initial velocity of the perturbed residue, respectively. The calculation results for these two cases are compared in Supplemental Table 1. It is found that the excitation times of the 11 residue clusters have only minor differences for these two cases. Based on the chronological order of the excitation of these residue clusters, the signaling pathways can be

identified, i.e. Asn127→cluster 4→cluster 9→cluster 5→cluster 8→cluster 7 (pathway 1) and Asn127→cluster 3→cluster 10→cluster 11→cluster 2→cluster 1 (pathway 2). The pathways identified by the method with non-zero initial velocity are the same as those in the case of zero initial velocity. Our results indicate that the initial velocity of the perturbed residue has no effect on the excitation order of the key residue clusters and the same energy transduction pathways were obtained.

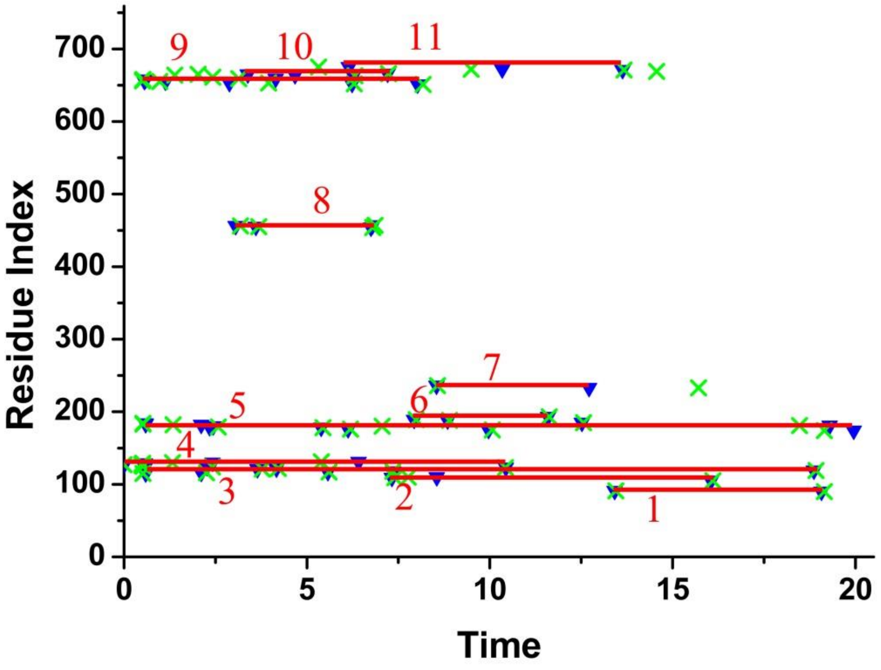

Supplemental Figure 1. The excited residues in myosin, whose squared fluctuation larger than 0.010 (in the case of zero initial velocity) or 0.011(in the case of non-zero initial velocity), along with their excitation times. The excited residues identified by the method with zero initial velocity are displayed in blue triangles, which are grouped into 11 clusters marked by the red lines along with the cluster numbers. The excited residues identified by the method with non-zero initial velocity are shown by the green crosses.

Supplemental Table 1. The excitation times of the 11 excited residue clusters obtained by the method with non-zero initial velocity compared with those in the case of zero initial velocity.

No. of residue clusters	Excitation times for zero initial velocity	Excitation times for non-zero initial velocity
1	13.420	13.447
2	7.327	7.400
3	0.591	0.517
4	0.001	0.001
5	0.590	0.517

6	7.931	7.986
7	8.543	8.570
8	3.052	3.186
9	0.553	0.501
10	3.385	2.026
11	6.150	5.318
